# Early Seizure Detection by Applying Frequency-Based Algorithm Derived from the Principal Component Analysis

**DOI:** 10.3389/fninf.2017.00052

**Published:** 2017-08-17

**Authors:** Jiseon Lee, Junhee Park, Sejung Yang, Hani Kim, Yun Seo Choi, Hyeon Jin Kim, Hyang Woon Lee, Byung-Uk Lee

**Affiliations:** ^1^Department of Electronics Engineering, Ewha Womans University College of Engineering Seoul, South Korea; ^2^Department of Neurology, Ewha Medical Research Institute, Ewha Womans University School of Medicine Seoul, South Korea; ^3^Department of Medical Science, Ewha Medical Research Institute, Ewha Womans University School of Medicine Seoul, South Korea

**Keywords:** electroencephalography, principal component analysis, seizure onset, early seizure detection, frequency-based feature

## Abstract

The use of automatic electrical stimulation in response to early seizure detection has been introduced as a new treatment for intractable epilepsy. For the effective application of this method as a successful treatment, improving the accuracy of the early seizure detection is crucial. In this paper, we proposed the application of a frequency-based algorithm derived from principal component analysis (PCA), and demonstrated improved efficacy for early seizure detection in a pilocarpine-induced epilepsy rat model. A total of 100 ictal electroencephalographs (EEG) during spontaneous recurrent seizures from 11 epileptic rats were finally included for the analysis. PCA was applied to the covariance matrix of a conventional EEG frequency band signal. Two PCA results were compared: one from the initial segment of seizures (5 sec of seizure onset) and the other from the whole segment of seizures. In order to compare the accuracy, we obtained the specific threshold satisfying the target performance from the training set, and compared the False Positive (FP), False Negative (FN), and Latency (Lat) of the PCA based feature derived from the initial segment of seizures to the other six features in the testing set. The PCA based feature derived from the initial segment of seizures performed significantly better than other features with a 1.40% FP, zero FN, and 0.14 s Lat. These results demonstrated that the proposed frequency-based feature from PCA that captures the characteristics of the initial phase of seizure was effective for early detection of seizures. Experiments with rat ictal EEGs showed an improved early seizure detection rate with PCA applied to the covariance of the initial 5 s segment of visual seizure onset instead of using the whole seizure segment or other conventional frequency bands.

## Introduction

Neuromodulation therapy such as vagus nerve stimulation (VNS), deep brain stimulation (DBS) or responsive neurostimulation (RNS) have recently been applied for patients with intractable epilepsy (Howland, 2014; Lee, 2014). In the closed loop neuromodulation system, which involves combined early seizure detection with automatic cortical stimulation, the accuracy of a seizure detection algorithm is crucial to improve therapeutic efficacy (Liang et al., 2013; Howbert et al., 2014; Moghim and Corne, 2014). Various methods for early seizure detection have been introduced, based on electroencephalographic (EEG) features such as frequency bands or magnitude variation (Guo et al., 2010; Howbert et al., 2014; Moghim and Corne, 2014). Especially, the selection of discriminative features is imperative for improving the accuracy and reliability of a seizure detection algorithm (Liang et al., 2010b).

To select discriminative features for accurate and reliable seizure detection, understanding of characteristics of seizure by phase change is a critical issue on early seizure detection. Epileptic seizure represents discriminative phase alteration over relevant time. In the previous studies, the epileptiform discharges and ictal rhythms were identified and analyzed over several window representatives of relevant time intervals during seizures. For examples, the electrographic seizures were partitioned into phases based on spatio-temporal evolution of ictal EEG phase changes (Niedermeyer and Lopes da Silva, 2005; Chaudhary et al., 2012). In another study, the ictal phases were divided into ictal onset, ictal established and late ictal periods (Thornton et al., 2010). The ictal onset phase was characterized by relatively prominent beta activity apparent. The late ictal phase was characterized by prominent gamma frequency band. In a more recent study, with the concept that the characteristics of epileptiform discharges could be distinguished even within several seconds during a seizure, the seizure dynamics was defined as the pre-seizure, the early seizure, the middle seizure, and the late seizure windows (Martinet et al., 2017).

Principal component analysis (PCA) can find a linear combination of frequency features with the maximum variance (Jolliffe, 1993), and is employed for EEG feature enhancement (Jolliffe, 1993; Liang et al., 2010a; Subasi and Ismail Gursoy, 2010; Yu et al., 2014) or EEG dimensionality reduction (Ghosh-Dastidar et al., 2008; Lekshmi et al., 2014; Siuly and Li, 2015) or de-noising (Kevric and Subasi, 2014). In a previous study (Ghosh-Dastidar et al., 2008), parameters were selected from the five physiological EEG subbands including delta, theta, alpha, beta, and gamma frequency ranges. The EEG subbands were then quantified in the form of the correlation dimension (CD), the largest Lyapunov exponent (LLE), and the standard deviation (STD). The PCA was employed for dimension reduction by transforming 9-dimensional feature spaces into a new feature space which was more amenable to subsequent EEG classification (Ghosh-Dastidar et al., 2008). In another study, the PCA was applied to 16 features (an approximate entropy and the powers of the 15 frequency subbands) and the resultant principle components (PCs) were fed to the classifiers for seizure detection. The PCA based features were regarded as the second feature type and the numbers of the PCs were determined based on the best performance of each classifier (Liang et al., 2010a). The above studies employed the PCA-based time-frequency EEG analysis to detect the seizure onset (Stafstrom, 2011; Perucca et al., 2013), which monitors frequency components of ictal EEGs (Guo et al., 2010; Subasi and Ismail Gursoy, 2010; Howbert et al., 2014). Other study described the effect of PCA as de-noising method in terms of epileptic seizure detection (Kevric and Subasi, 2014). They applied PCA de-noising method to EEG segments prior to Power Spectral Density (PSD) estimation. Although PCA is not a novel method, it is one of the conventional methods that has been widely used and tested to validate the accuracy for seizure detection. Besides, PCA is an easy and fast method that can be an important merit to develop as a real-time analysis method.

In the present study, we used a frequency-based algorithm derived from PCA to extract features of EEG changes during seizures. The main purpose of this study was to extract a representative EEG feature for seizure onset from all frequency bands to improve the performance. In addition, we compared the results between EEG features from early segment of seizure vs. those from the whole seizure segment. These two issues are one of the most important parts in seizure detection regardless classification method are used for this purpose. So, in this study, we wanted to test these fundamental elements related to seizure detection, rather than developing another new analysis technique. For this purpose, it would be better to use a straight forward method rather than a new method that has not been verified yet.

**Figure 1:**
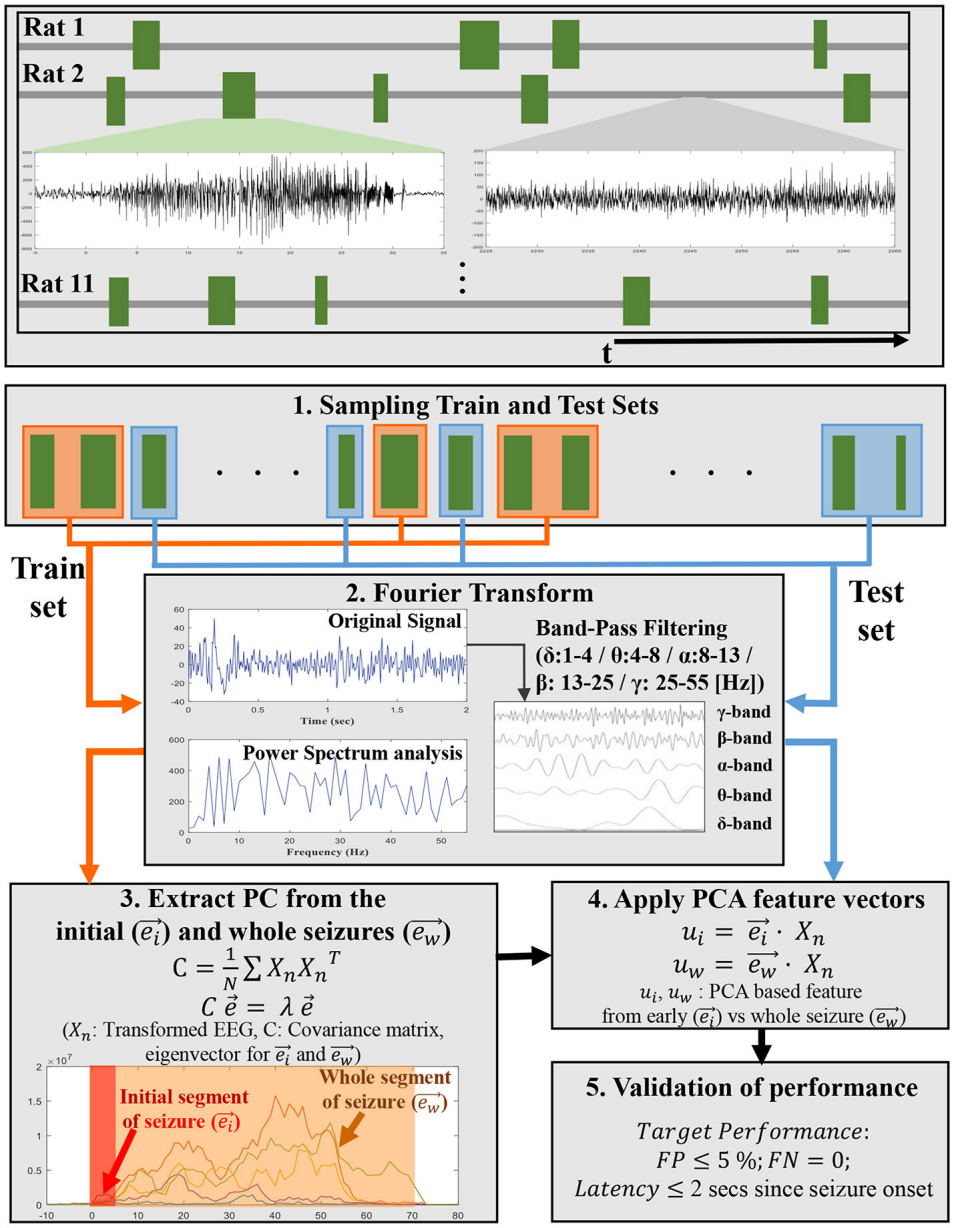
A schematic flowchart shows an overview of the steps involved in developing PCA-based EEG features in each subband energy from ictal EEGs in training set and application for early seizure detection to ictal EEG segments in test set. The top panel shows ictal EEGs trains for 11 pilocarpine-induced epileptic rats. The mid-left plot in the top panel represents seizure signal and the mid-right plot in the same panel represents non-seizure signal. The random samplings for Train and Test sets are displayed by orange and blue line. In step 3 and step 4, $\vec{e_{i}}$ and $\vec{e_{w}}$ represent principal component (PC) vector extracted from initial segment of seizures and whole seizures, respectively.

We applied the PCA in terms of the frequency-energy distribution of sub-frequency bands (Figure 1). We compared the energies of various frequency bands and additional features with different weights on these frequency bands depending on how the specific frequency bands change during seizures over time. To this end, we proposed employing a dominant eigenvector with the maximum eigenvalue from the covariance matrix to characterize the ictal EEG changes. In addition, we compared the eigenvectors for the whole segment of seizures and only the initial segment of seizures to capture the characteristics of seizure patterns. Interestingly, we could detect the seizure onset even more accurately by using the covariance of the initial segment of seizures, especially the initial 5 s from the seizure onset when compared with the whole seizure segments. This study contributes to improve the accuracy of early seizure detection algorithm by extracting discriminatory features from the EEG signals during seizures, especially from the initial segment of ictal EEG seizure rather than the whole seizure segments.

## Methods

### Animal preparation and seizure recording for analysis

A total of 15 adults (4 weeks old) male Sprague-Dawley rats (Orient: KOATECH, Gyeonggi-do, Korea) weighting 280–300 g were used initially for this study. Before the surgery, all rats were in a controlled environment with a 12-h light-dark cycle and the temperature was set at 22–23°C. The experimental animals could freely access food and water. The rats were anesthetized with a mixture of ketamine (80 mg/kg) and xylane (5.2 mg/kg), and the skull was drilled after a scalp incision with the animal's head placed on a stereotaxic frame for epidural electrode insertion. Two epidural electrodes were placed on the left and right central cortical C3 and C4 regions using One Channel Electrode System with Ground Preclinical Component (PlasticsOne, Inc., Roanoke, VA, USA), respectively (AP = 3, ML = ±3.5). Additional two screw electrodes were fixed to the frontal and parietal bone as the reference and ground, respectively. After surgery, the rats were left to recover for 1 week before the EEG recording, which was carried out in a separate cage. One animal died during the anesthesia and another died after the surgical procedure.

All animals were administered with pilocarpine hydrochloride (300–380 mg/kg i.p., Sigma-Aldrich) for inducing seizure as described previously (Turski et al., 1983; Hamani and Mello, 2002; Kobayashi et al., 2003; Kumar and Buckmaster, 2006; Kwak et al., 2006; Curia et al., 2008). Diazepam was received 8.6 mg/kg (Sigma-Aldrich) depending on the seizure severity after 120 min of initial status epilepticus (SE) to reduce the mortality rate and to minimize suffering from seizure. Additional diazepam was provided 30–40 min later if the SE had not stopped, and the animals were included for long-term recording. Two further animals died during the process of inducing SE and valium injection. Finally, the remaining 11 animals were included for long-term recording. Euthanasia was determined at the end of the EEG recording. After the end of monitoring, we performed euthanasia using a carbon dioxide (CO_2_) chamber. This study was approved by the Ethical Committee for Animal Investigation of the Ewha Womans University School of Medicine, and the protocol was carried out in accordance with the recommendations of the National Institutes of Health (NIH) Guidelines for the Care and Use of Laboratory animals.

For long-term monitoring, all rats were placed individually in custom-made 15 × 40 × 25 cm Acryl cages. Non-seizure EEG signals for normal conditions were recorded for 2 h before the pilocarpine injection. The EEG amplifier systems contained separate ground and reference inputs using the Grass Telefactor long-term EEG monitoring system (the Grass Technologies Inc., West Warwick, RI, USA). A total of 4 EEG channels were acquired using 16-bit A/D conversion, 200 Hz sampling frequency (fs), 90 dB common mode rejection ratio, and a 0.1–70.0 Hz band pass filter.

All of the 11 surviving animals developed the initial SE and later spontaneous recurrent seizures (SRS), which were monitored for 24 h per day for up to 6 weeks after the pilocarpine injection by using a continuous video-EEG monitoring system (Twin 7.0, Grass-Telefactor, West Warwick, RI, USA). Approximately 1–2 weeks after the initial SE, the rats developed SRS. A total of 100 ictal EEG segments from 11 epileptic rats that survived were chosen for inclusion in this study. First, the entire recordings after the initial 7 days of pilocarpine injection were reviewed to identify ictal EEG segments for SRS by experienced neurologists (HWL, HJK) and neuroscientist (YSC). The onset and offset timing of ictal EEGs during SRS were determined by using traditional visual analysis methods. Criteria for selection included: (i) at least 4 weeks of prolonged EEG recordings with good quality after pilocarpine injection, (ii) prolonged EEG recordings in which one or more seizures were captured, (iii) at least 10 s of total seizure duration for comparison of the initial 5-s seizure segments and the whole seizure segments, and (iv) at least stage 3–5 seizures according to the modified Racine's classification (Racine, 1972). For each animal, up to the first 10 seizures were included initially, and ictal EEG segments that had movement or electrode artifacts were excluded by visual inspection, finally giving a total of 100 ictal EEG segments remained and included for the further analysis. Among the final 100 ictal EEG segments, 50 segments were chosen randomly as training dataset to minimize possible bias during selection, and the remaining 50 segments were used for validation of the proposed PCA method as test dataset. The ictal EEG segment for each seizure was clipped from 60 s before the visual seizure onset until 60 s after the visual seizure offset. The electrode impedance was <10,000 Ω for each electrode.

### PCA to extract EEG features for seizure onset detection

#### Subband partition of EEG

In this research, a total of 100 ictal EEGs were partitioned into 50 training sets and 50 testing sets. After we identified epileptic seizure segments, samples for train and test process were randomly sampled to minimize any bias during selection.

**Figure 2:**
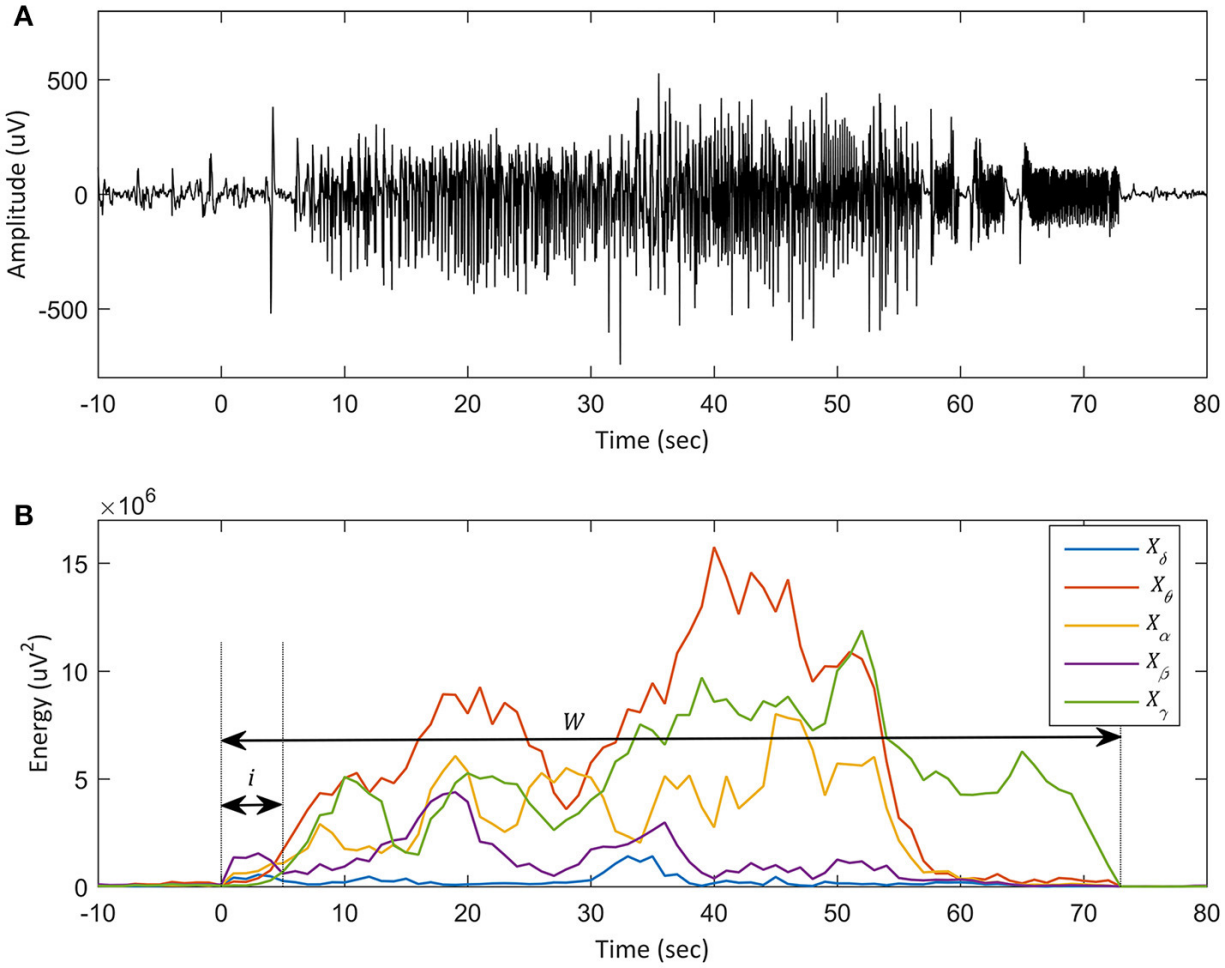
Plot of an ictal EEG in training set and each subband energy. **(A)** Plot of the ictal EEG, seizure No. 1 in the training set. The x-axis denotes time (sec), and the y-axis represents the amplitude of the iEEG signal. Onset time is defined as *t* = 0. **(B)** Plot of energy distribution of the same seizure in each EEG subband. The y-axis represents the amplitudes of subbands and the x-axis denotes time. The five subbands are derived from one recording channel. The blue line, bold red line, yellow line, bold purple line, and green line indicate the energy of the delta (δ: 1–4 Hz), theta (θ: 4–8 Hz), alpha (α: 8–13 Hz), beta (β: 13–25 Hz), and gamma (γ: 25–55 Hz) frequency bands, respectively. In **(B)**, the short black arrow represents the initial 5 s of seizure (i) and the long black arrow represents the whole segment of the seizure (w). The prominent change of energy in the beta band can be observed in the initial segment of the seizure. In the latter portion of the seizure, the energy of the theta and beta band is dominant. Thus, for early detection of the onset, it is more accurate to use a feature that has more weight on the beta band.

The key steps for the analysis was outlined in Figure 2. The EEG signal was transformed to the frequency domain using the fast Fourier transform (FFT), and was then partitioned into five conventional EEG frequency bands: delta (δ: 1–4 Hz), theta (θ: 4–8 Hz), alpha (α: 8–13 Hz), beta (β: 13–25 Hz), and gamma (γ: 25–55 Hz) ranges. In this research, the delta band excluded 0–1 Hz to avoid offset noise and the gamma band was included up to 55 Hz to eject AC line power frequency noise of 60 Hz (Watrous et al., 2011; Sharif and Jafari, 2017). The time window for FFT analysis was 2 s. We represented time domain EEG data as x(n). Since the magnitude of ictal EEG tends to be inversely proportional to the frequency (Osorio et al., 1998), this tendency was overcome using a simple difference filter as shown in Equation (1), and the signal was then transformed to the frequency domain:

(1) $$\begin{array}{r} X\left[k\right]=\overset{N-1}{\underset{n\;=\;0}{\sum }}\left[x\left(n+1\right)-x\left(n\right)\right]e^{-j\frac{2\pi }{N}kn}\;\left(0\leq k\leq N-1\right), \end{array}$$

where *X*[*k*] is the Discrete Fourier Transform of EEG after passing the difference filter, and *N* is the number of samples in the window, which is fs^*^2 s = 400. From the transformed EEG *X*[*k*], the signal energy of five subbands was obtained as shown in Equation (2):

(2) $$\begin{array}{r} X_{subband}=\overset{f_{e,subband}}{\underset{f_{b,subband}}{\sum }}{\left|X\left[k\right]\right|}^{2},\;subband\;\in \;\left\{\delta ,\;\theta ,\;\alpha ,\;\beta ,\;\gamma \right\}. \end{array}$$

The vector of the five subbands frequency band energies, *X*_δ_, *X*_θ_, *X*_α_, *X*_β_, and *X*_γ_, at time *n* was represented as vector *X*_*n*_:

(3) $$\begin{array}{r} X_{n}={\left[X_{\delta n}\;X_{\theta n}\;X_{\alpha n}\;X_{\beta n}\;X_{\gamma n}\right]}^{\text{T}}\;\left(1\leq n\leq W\right). \end{array}$$

In Equation (3), *W* is the number of windows of which the size was 2 s with a 1 s overlap. Each component of *X*_*n*_ represents the energy of each subband during the 2 s window. The original signal and energies of each frequency band in seizure No. 1 are plotted in Figure 2. Immediately after seizure onset, the β band showed an obvious increase followed by the α, δ, θ, and γ band powers. In the mid-to-late seizure, unlike the initial segment, the θ band power revealed a significant increase, followed by the γ, α, and remaining β or δ band powers.

#### Principal component as a feature vector

The PCA was applied to the covariance matrix of 50 ictal EEG training seizures using the Statistical Toolbox and Signal Processing Toolbox in Matlab 8.2 (MathWorks, Natick, MA, USA). With each threshold obtained from the random sampled training set, we compared the performance of the seven feature vectors on the test seizures for validation of the PCA method: two PCA based features from the initial and the whole segment of seizures as well as the energy from each of the five subbands in the testing set with 50 seizures chosen randomly. The covariance matrix $C=\frac{1}{N}\sum {X_{n}X_{n}}^{T}$ was calculated from the training seizure signal vector, *X*_*n*_. The covariance matrix is a summation over 50 training seizures. A feature vector, eigenvector $\vec{e}$, combined with eigenvalue λ was then obtained from Equation (4):

(4) $$\begin{array}{r} C\vec{e}=\lambda \vec{e} \end{array}$$

The dominant eigenvector with the maximum eigenvalue, $\vec{e_{d}}$ was selected as a feature for onset detection. The eigenvector from covariance matrix C represented the principal direction of the ictal subband energies. In this experiment, the PCA based feature *u* for seizure detection was obtained by the dot product of *X*_*n*_ and $\vec{e_{d}}$ (Equation 5), which was the energy component along the major ictal energy direction $\vec{e_{d}}$. Here, the directions of the non-seizure vector differed from those of the first principal component vector of the ictal energy; therefore, the feature signal for the non-seizure was quite small, which reduced FP significantly.

(5) $$\begin{array}{r} u_{n}=\;\vec{e_{d}}\cdot \;X_{n} \end{array}$$

The rhythmic activities of the seizures varied with the progression of time (Stafstrom, 2011; Perucca et al., 2013). Therefore, the initial segment of seizure immediately after the onset revealed a quite different frequency-energy distribution from the latter phase of the seizure. For further analysis in our study, we compared the combined EEG features of frequency-energy between the early seizure vs. the whole seizure segments. Since the goal of our work was the extraction of features for early detection of a seizure, it was essential that the feature could capture those characteristics of the initial segment of seizures that were distinct from the latter phase. We adopted an eigenvector with the maximum eigenvalue of the covariance matrix from the initial segment of training seizures as a feature for onset detection, and verified the effectiveness of the proposed feature using testing seizures. We confirmed the efficacy of the proposed feature from experiments.

**Figure 3:**
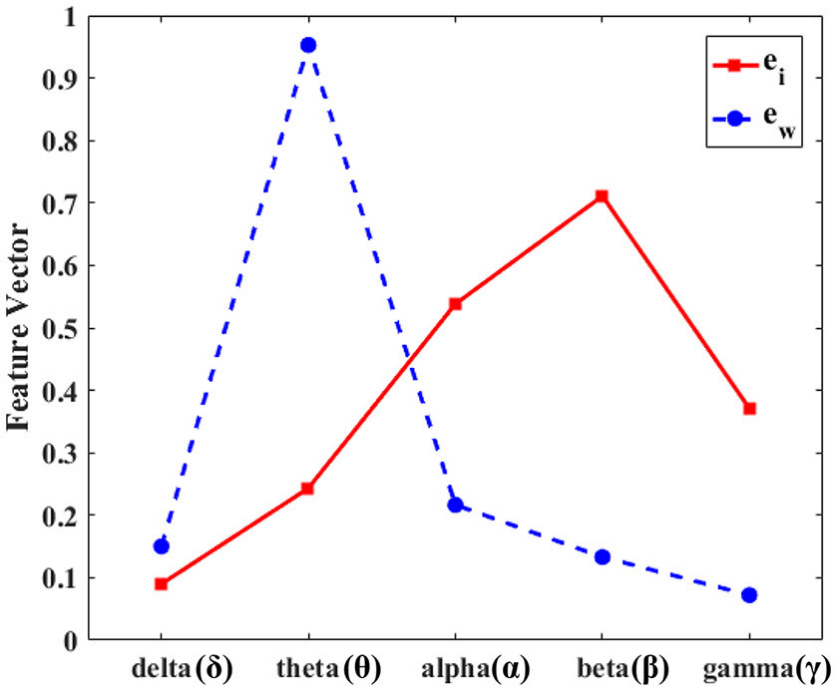
Dominant eigenvectors from the initial segment vs. the whole seizure segment. The dominant eigenvector from the principal component analysis for the initial 5 s after the seizure onset ($\vec{e_{i}}$) vs. the whole segment ($\vec{e_{w}}$) of seizures. The y-axis represents the normalized values of the dominant eigenvectors and the x-axis denotes the five subbands. The red line is the eigenvector of the initial 5 s and the blue dashed line represents the eigenvector of the whole seizure segment. The beta frequency band is dominant in the initial segment of seizure (red line), while the theta frequency is strong during the whole seizure segment (blue dashed line). The two feature vectors, $\vec{e_{i}}$ and $\vec{e_{w}}$, show quite different tendencies.

We set a feature vector $\vec{e_{w}}$ to be the feature vector extracted from the whole segment of seizures, and set $\vec{e_{i}}$ to be the proposed feature using the initial 5 s of seizures in the training set. We compared the angle between the two feature vectors $\vec{e_{i}}$ and $\vec{e_{w}}$ as shown in Figure 3; the angle would be zero if the feature vectors had identical characteristics. The angle between the two vectors was derived from a dot product from Equation (6):

(6) $$\begin{array}{r} \vec{e_{w}}\cdot \;\vec{e_{i}}=||\vec{e_{w}}||\;||\vec{e_{i}}||\cos\theta \end{array}$$

As shown in Figure 3, the two feature vectors, $\vec{e_{i}}$ and $\vec{e_{w}}$, showed quite different tendencies; the angle between the two feature vectors was 33.1°. The weight on the theta band was dominant in $\vec{e_{w}}$ which is from the whole ictal EEG segment, while the weight on the β band was dominant in $\vec{e_{i}}$. Dot products calculated from each of these feature vectors became the PCA based feature for seizure detection, *u*_*i*_ and *u*_*w*_. With these two PCA based features and the energy of the 5 subbands, we tested whether PCA based features were more effective for early detection of seizures, and especially for the application of the proposed feature, PCA, on the initial segment of seizures: the proposed PCA feature showed better performance than that on the whole segments of seizures.

### Estimations of thresholds, false positive, and false negative

**Figure 4:**
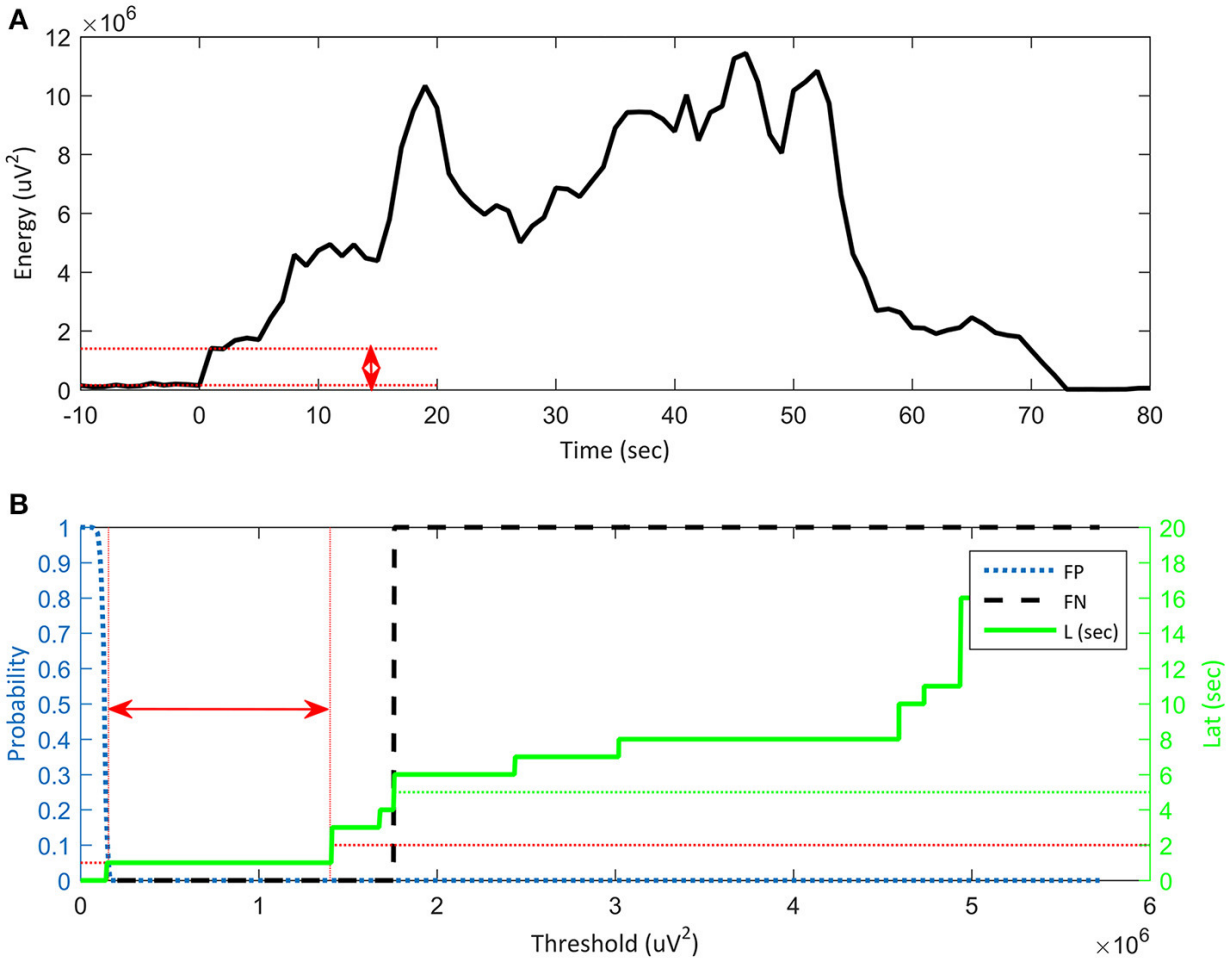
Reliability of algorithm based on false positive (FP), false negative (FN) and latency vs. threshold. **(A)** Threshold range (red arrow) for stable seizure detection on a plot of the features. Onset time is defined as *t* = 0 s. The y-axis represents the energy of a feature and the x-axis denotes time. **(B)** Energy threshold range (red arrow) for stable seizure detection in terms of standard accuracy indices: Lat ≤ 2 s, FN = 0 s and FP ≤ 5%. The x-axis represents a threshold that is the same as the energy of the feature in **(A)**. The left y-axis represents the probability of FP: 0.05 is 5% and 1 is 100%. The blue dotted curve shows FP, which decreases with increasing threshold. The right y-axis denotes time in seconds and the green line represents latency. Latency increases with increasing threshold. When the latency is greater than 5 s (green dotted line), it is classified as FN, as shown by the vertical black dashed line. The threshold range for stable seizure detection is shown as the red bidirectional horizontal arrow, which is the range of the feature threshold from EFP = 0.05 to ELat = 2. The wide threshold range means that the feature has a sufficient noise margin, which guarantees reliable onset detection.

We adopted a straightforward thresholding algorithm for an intuitive comparison of various features in onset detection, because the purpose of this work is to select an effective feature for seizure detection instead of implementing a detector. While it was known that employing a sophisticated classifier can enhance the accuracy, a simple thresholding algorithm manifested the comparison of different performances among the features. We used quantitative metrics such as false positive (FP), false negative (FN), and latency (Lat), as statistical measures to test reliability of performance for this kind of EEG analysis method (Singh and Aktas, 2016). Lat was defined as the time delay of the detected onset using a feature from the onset defined by neurologists. Our aim for seizure detection was to apply a therapeutic pulse before a “significant symptom” manifested; therefore, we limited the Lat to 5 s. Thus, a seizure which was not detected within 5 s was classified as FN. FN in this study was the ratio of undetected seizures to the total number of test seizures. FP referred to a portion of misjudged non-seizure signals as seizures. Non-seizure samples with total duration 1.5 h were used to test for the FP. The number of non-seizure windows of which the feature amplitude was greater than a given threshold was counted as FP. FP decreased as the threshold increased. FP is represented by a blue line in Figure 4B.

The thresholds were determined from median values of thresholds of training seizures. Range (red arrow in Figure 4B) for target performance is as follows: FP was less than 5%, FN is zero, and Lat was less than a specified value of 2 s (Figure 4A). FP decreased with increasing threshold; therefore, the threshold must be high enough to maintain a low FP (5% in our experiment), which was denoted as EFP = 0.05. On the other hand, FN and Lat increased with increasing threshold. Therefore, the threshold should be low to avoid FN. Lat was a crucial measure for onset detection. If Lat was longer than 5 s, it was classified as FN as shown in the lower graph of Figure 4B. The threshold for a specified latency, for example 2 s, was represented as ELat = 2. The arithmetic average of thresholds for EFP = 0.05 and ELat = 2 was a good candidate for threshold for a given training seizure. We then selected the median among the thresholds of the training seizures.

### Additional validation for comprehensive data analysis

For further comparison to improve validity, we additionally tested the rate of FP during 5-days of non-seizure data (425,100 s) to verify the proposed method. The non-seizure data included not only interictal spikes, but also moving artifacts of rats and electrical artifacts (Supplementary Figure 1). The non-seizure samples were taken between SRS at least 1 h before and after seizures. In Supplementary Figures 1A,B show interictal spikes, while Supplementary Figures 1C,D represent electrical or moving artifacts. The detected feature is normalized against the corresponding threshold value, therefore, magnitude over 1 means FP. In comparison, the proposed feature with low value less than 1 means minimal FP.

## Results

Feature extraction and testing for seizure detection were performed with 100 ictal EEG events: 50 ictal EEGs for training and the remaining 50 for the test. To elucidate whether the size of training dataset had some effects on the proposed algorithm, we compared the PCA features derived from the initial seizure and the whole seizure segments using different training dataset of 10, 20, 30, and 50 ictal EEGs. The average of the PCA-based eigenvectors showed similar characteristics regardless of the sample size; however the standard deviation increased as the sample size decreased. When compared the eigenvectors from the initial seizure vs. the whole seizure segments, the discrepancy of the PCA vectors according to the size of training dataset from the latter was more evident. To be specific, the overall trend of eigenvectors from the initial seizure segments showed similar findings across different numbers of the training dataset, that is, the highest weight was on the beta band followed by the alpha band and the lowest weight was on the delta band. On the other hand, those from the whole seizure segments revealed that the larger numbers of the training dataset, the more evident characteristics of the weight on the five subbands were, especially in the theta and beta bands (Supplementary Figure 2). PCA was applied to the covariance matrix of the training seizures. The covariance matrix was constructed from the initial segment of seizures and then from the whole segments for comparison. From each of the two covariance matrices, we obtained two PCA based feature vectors, $\vec{e_{i}}$ and $\vec{e_{w}}$. The two PCA results were used as the feature vector in the testing set. The seven features, including *u*_*i*_ and *u*_*w*_, were compared from two perspectives: (i) the standard accuracy index (FP, FN, and Lat) and (ii) the threshold range for stable seizure detection.

### Standard accuracy indices: FP, FN, and Lat vs. thresholds

**Figure 5:**
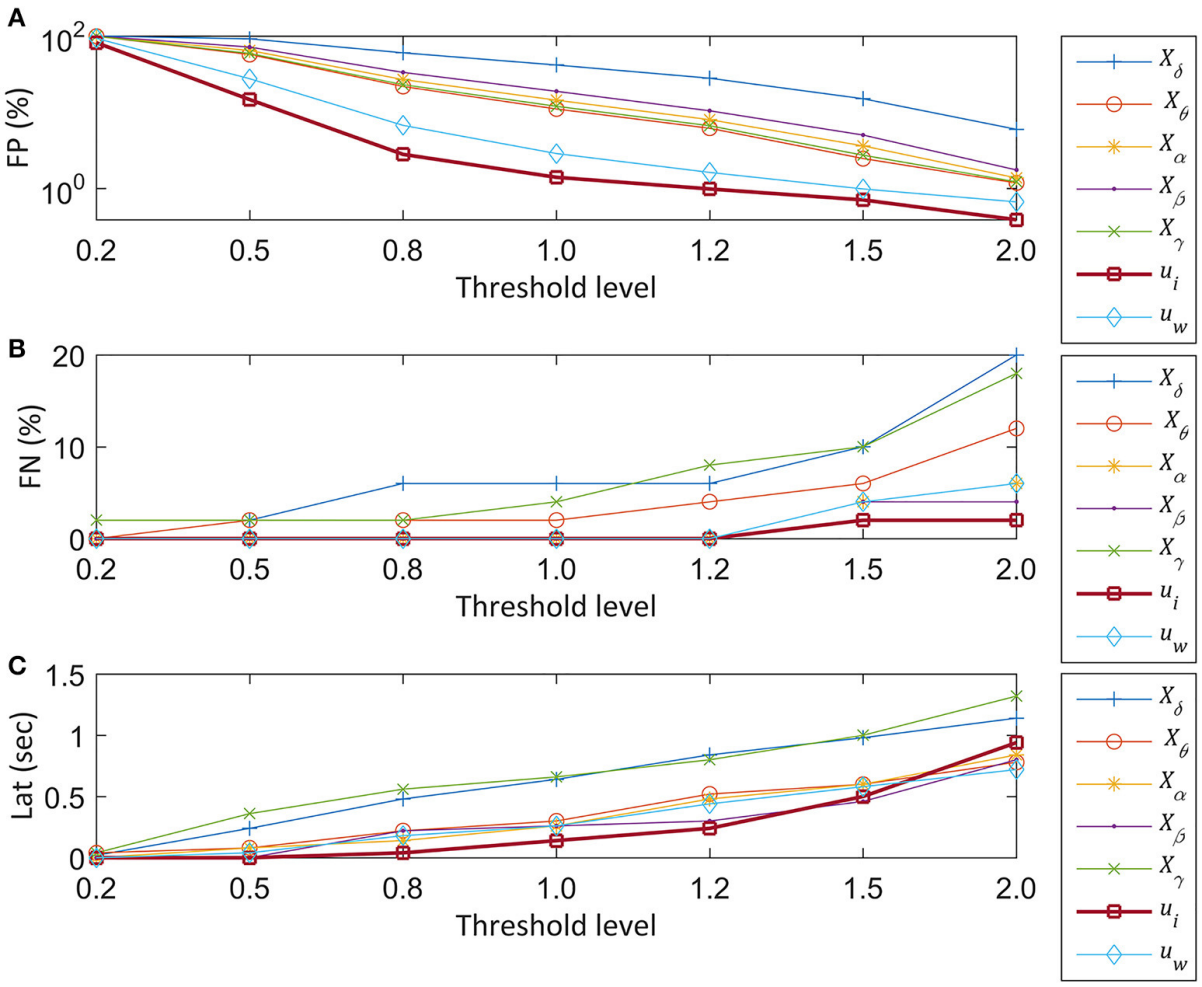
Performances of seven EEG features vs. threshold levels. The normalized median threshold (*Th*_1_) and scaled thresholds at 0.2, 0.5, 0.8, 1.2, 1.5, and 2 times of the normalized median value are compared for seizure detection. The sky-blue plus sign-pointed line, orange circle-pointed line, yellow star-pointed line, purple dot-pointed line, green cross-pointed line, bold blue square-pointed line, and bold red diamond-pointed line indicate the delta (δ), theta (θ), alpha (α), beta (β), gamma (γ) band features, the PCA based feature from the initial segment of the seizure segment (*u*_*i*_), and the other PCA based feature from the whole seizure (*u*_*w*_), respectively. **(A)** Plot of FP at the scaled *Th*_1_. The *Th*_1_ is derived from the training set. The y-axis represents FP from a test set and the x-axis represents the different scales of the *Th*_1_. **(B)** Plot of FN vs. the threshold. The y-axis represents the FN in percentage, and the x-axis represents the threshold. **(C)** Plot of Lat vs. threshold change.

The standard accuracy indices were investigated for each feature at the median threshold (*Th*_1_) obtained from the training set. In addition to the threshold, we measured the performance indices after changing the threshold to 0.2, 0.5, 0.8, 1.2, 1.5, and 2 times the median value. The results for the standard accuracy indices are shown in Figure 5. The horizontal axes showed the threshold change and the vertical axes showed FP (%), FN (%), and Lat (sec). The threshold values are normalized to 1 in the plot. We observed that the higher threshold results in the lower FP as shown in Figure 5A. The proposed feature *u*_*i*_ showed the lowest FP of all threshold ranges, followed by the other PCA based feature, *u*_*w*_: FP of *u*_*i*_ is 1.4% and FP of *u*_*w*_ is 2.9%. The theta band feature showed the worst performance with 41.7% FP at the median threshold.

The FN increased with increasing threshold (Figure 5B). The FN was zero, regardless of the threshold change from 0.2 to 1.2 for four features: θ, β, *u*_*i*_, and *u*_*w*_. However, when the threshold was increased by 1.5 times, FN of the other features increased further than the proposed PCA feature, *u*_*i*_. The δ and γ band features showed worse performance within the given range of threshold change. The expected tendency of the higher Lat with higher threshold was observed in Figure 5C. For the normalized range from 0.2 to 1.2, the Lat of *u*_*i*_ showed the smallest latency time. For example, at the threshold 1, Lat of *u*_*i*_ was 0.14 s, followed by Lat of *X*_α_ of 0.26 s. In contrast, the θ and γ band features showed longer latency than the other subband features, which is similar to the tendency of FN.

### Threshold range for stable seizure detection

**Figure 6:**
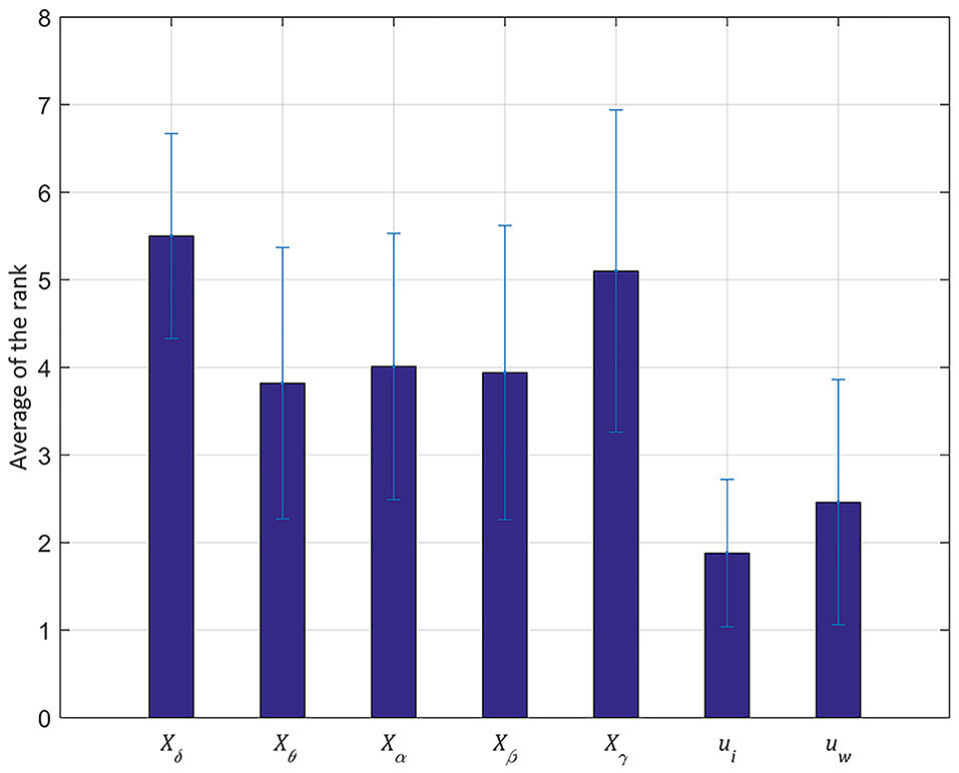
Average of the rank for stable seizure detection in a testing set. The y-axis represents the average of the ranks and the x-axis denotes the seven features. The average is calculated from the 50 testing seizures and the standard deviation is represented by a black line. The principal component from the initial segment of seizures, *u*_*i*_, performs the best rank among all the seven features with relatively low false posivie (FP) and latency (Lat).

**Figure 7:**
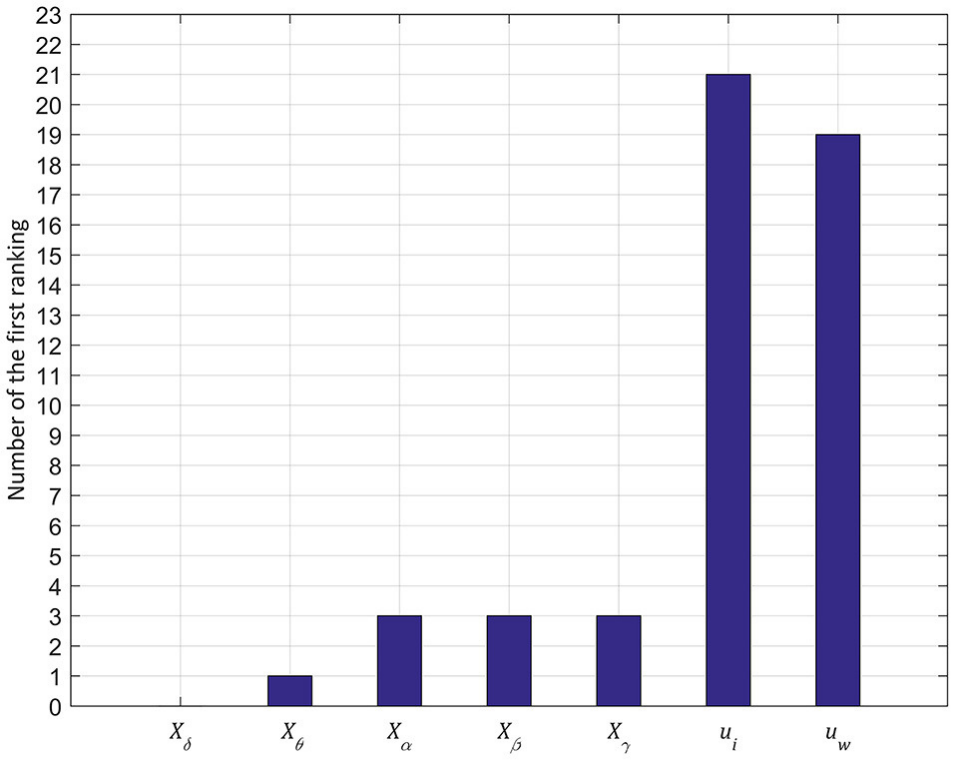
Number of first ranking in 50 testing seizures. The y-axis represents the number of first rankings among the 50 testing seizures and the x-axis denotes the seven features. Similar to the result of the average of the rank in Figure 6, the principal component from the initial segment of seizures, *u*_*i*_, performs the best ranking among all the seven features with relatively low FP and Lat.

From these standard accuracy indices, we can determine the range of threshold values. The range of threshold values is also crucial for the stable operation of seizure detecting devices (Shoeb and Guttag, 2009). Thus, we estimated the range of threshold values for each feature in all test seizures and then determined the ranking of widths. Figure 6 shows the average ranking of all test seizures for each feature. The PCA based feature, *u*_*i*_, was ranked highest with 1.88 ± 0.84 among the seven features, followed by another PCA based feature, *u*_*w*_, with 2.46 ± 1.40. The θ band feature, *X*_δ_, was ranked lowest with 5.5 ± 1.17 and the γ band feature, *X*_γ_, ranked sixth with 5.1 ± 1.84. Overall, the worse performance in the θ and γ band features has a similar tendency to the result of 3.1 as shown in Figure 5. Moreover, we counted the number of first ranks for each feature as shown in Figure 7. In Figure 7, the x-axis shows the seven features and the y-axis shows the number of the first rank.

Consequently, *u*_*i*_ had the greatest number of first rankings with 21 among 50 test seizures. The *u*_*w*_ followed *u*_*i*_ and the rest of the features had fewer than 3 times for the first rank. From the result, the two PCA based features showed a more flexible threshold range than the other features; especially, the PCA based feature derived from the initial segment of seizures, *u*_*i*_, showed the highest stability. In conclusion, with these two experiment results, we confirmed that *u*_*i*_ revealed the best performance among all the seven features with relatively low FP and Lat, which will guarantee safe operation margins in early detection of onset.

### Examples of non-seizure segments detected as false positives

Supplementary Figure 1 shows examples of artifacts; interictal spikes (A,B), electrical artifacts (C) and moving artifacts (D). The detected feature has been normalized against the corresponding threshold value, therefore, magnitude over 1 means false positive. The proposed feature, *u*_*i*_ maintains low value less than 1, which means FP was minimal. The proposed PCA feature during interictal spikes, electrical and moving artifacts shows low magnitude less than 1 for most of the time.

Low FP results for artifacts also support the superiority of the proposed feature in comparison with other features. Supplementary Table 1 shows the rates of FP for the additional data for 5 days including non-seizure data including artifacts in the initial and the whole seizure segments. The rate of FP for the proposed feature, *u*_*i*_, was quite low and comparable between the initial and the whole seizure segments.

## Discussion

In the present study, we investigated the performance of seizure features for early detection. The main findings of this study were: (i) the PCA based feature based on weighted average of five EEG frequency bands was better in detecting the seizure onset than the individual frequency band, (ii) the PCA feature from the initial segment of seizures showed higher accuracy than that from the whole segment of seizures. Feature extraction by PCA algorithm and the features extracted from five EEG frequency bands have been used in previous researches. However, the main purpose of this study was to investigate characteristics and usefulness of the features extracted from the initial part of the seizures for early seizure detection compared with those from the whole seizures. Our findings indicated that PCA is useful for extracting the feature of seizures and PCA from the initial segment of ictal EEG could detect a seizure more accurately than the principal component from the whole seizure segments. The FP of the proposed method was 1.4%, while the second best was 2.88% using PCA of the whole seizure segments. Lat was 0.14 s, and the second-best performance was 0.26 s using the alpha channel.

In previous studies, PCA was applied to the whole segment (Srinivasan et al., 2005; Kevric and Subasi, 2014) or dimensionality reduction (Polat and Güneş, 2008; Subasi and Ismail Gursoy, 2010; Yu et al., 2014). In a study (Ghaffari and Ebrahimi Orimi, 2014), wavelet packet transform (WPT) was used in each of the frequency bands, while the energy and entropy functions were composed of the wavelet coefficients and used as feature vectors. After feature selection, 15 energy and entropy features were selected as the final features for seizure detection. However, with the wavelet-analysis, the infinite number of wavelets and the width of the scaling function should be circumvented (Valens, 1999).

From our results, we verified that the PCA based feature vector extracted from the initial 5 s since the seizure onset outperformed the frequency subband as well as the PCA based feature vector extracted from the whole segment of seizures. The two feature vectors for the initial seizure ($\vec{e_{i}}$) and the whole seizure ($\vec{e_{w}}$) segments, showed different characteristics in terms of energies in different frequency bands. The weight on the theta band was dominant in $\vec{e_{w}}$, while the weight on the β band was dominant in $\vec{e_{i}}$. In fact, the strong weight on the β band in $\vec{e_{i}}$ is more comparable to the previous findings (Chaudhary et al., 2012). Thus, the initial segment of seizures seems to be good for extracting characteristic features during seizures, which can be advantageous for real-time EEG recording for early seizure detection. One possible explanation for the difference in the frequency band energy distribution of the initial segment of seizures from the latter part of the seizures was rhythmic build-up and/or propagation of seizure activities. Other studies also demonstrated that various EEG indices showing EEG rhythms and functional networks revealed different patterns at the seizure onset, during seizure propagation, and at seizure termination (Kramer et al., 2010, 2012; Kramer and Cash, 2012). The initial segment of seizures revealed prominent and robust changes, and these characteristics could be encoded in the PCA. Therefore, the proposed feature could detect the seizure onset with higher accuracy while maintaining low latency. Thus, once the seizure detection algorithm with our PCA based feature was embedded in a medical device such as a real-time seizure detector, we could anticipate improvement of therapeutic efficacy with better performance. The proposed algorithm requires a pre-computation of eigenvectors from training seizures. This eigenvector has five weighting factors for the corresponding subbands, and the feature for seizure detection is a weighted average of five subbands of the EEG signal. Therefore, the overhead for feature calculation is five multiplications and four additions, which are negligible compared to the subband division of the EEG signal. In this study, we additionally selected 5 days of non-seizure EEG signals including interictal spikes and electrical artifacts (425,100 s) to verify the proposed method. Results of the FP from these artifact samples further validated that the proposed feature showed a reliable FP rate comparable between in the initial and the whole seizure segments.

Future works include focusing on applying the feature to more elaborate classifiers. The proposed PCA feature could be applied to other methods such as Support Vector Machine (SVM) for robust and precise detection of seizure onset. A patient-specific SVM can improve the performance of neuro-stimulators (Shoeb and Guttag, 2009) and another study compared the performance of three SVM types: weighted SVM, one-class SVM, and support vector data description (SVDD) for seizure detection in an animal model of chronic epilepsy (Nandan et al., 2010). Therefore, with the proposed feature and patient-specific classifier such as SVM, we can anticipate the improved performance in early seizure detection. Moreover, this feature could be used for a closed-loop system with a therapeutic pulse, because of the robust and accurate detection of the seizure onset.

In summary, we observed that the PCA based features applying different weights for various frequency bands, especially from the initial segment within 5 s after the seizure onset were effective for early seizure detection for ictal EEG events of spontaneous seizures in the Pilocarpine-induced rat epilepsy model. This research can contribute to real-time seizure detection, in which the EEG characteristics are analyzed in clinical settings where the timing of seizure detection is crucial for aborting the seizure activities. The presented PCA feature derived from the initial segment of seizures is simple and fast in the calculation point of view, which can be adaptable to the future clinical application of real-time EEG monitoring for early seizure detection. Therefore, the detection electronic circuitry can be designed to consume less power for seizure feature estimation. Our findings could enable us to develop a robust and accurate algorithm for automatic and real-time early seizure detection in future studies. With this approach, advanced closed loop neuro-stimulation methods for patients with drug-resistant intractable epilepsy can be implemented with further validation.

## Author contributions

JL, JP, HWL, and BUL interpreted data, conceptualized and designed the study. HK and YC acquired the raw data. JL, JP, SY, YC, and HJK analyzed the data. JL and JP drafted, and HWL and BUL revised the work with important intellectual content.

### Conflict of interest statement

The authors declare that the research was conducted in the absence of any commercial or financial relationships that could be construed as a potential conflict of interest.
