# Supplementary material for: Early Seizure Detection by Applying Frequency-Based Algorithm Derived from the Principal Component Analysis
Source: Front Neuroinform. 2017 Aug 17;11:52. doi: 10.3389/fninf.2017.00052 (PMC5562675; doi:10.3389/fninf.2017.00052)
Supplement: Supplementary file 1 [file Table1.DOCX]

| **Feature**  **False  Positive** | **Delta (**$\boldsymbol{X}_{\boldsymbol{\delta}}$**)** | **Theta (**$\boldsymbol{X}_{\boldsymbol{\delta}}$**)** | **Alpha (**$\boldsymbol{X}_{\boldsymbol{\delta}}$**)** | **Beta (**$\boldsymbol{X}_{\boldsymbol{\delta}}$**)** | **Gamma (**$\boldsymbol{X}_{\boldsymbol{\delta}}$**)** | **PCA based feature from** $\vec{e_{w}}$ **(**$\boldsymbol{u}_{\boldsymbol{w}}$**)** | **PCA based feature from** $\vec{e_{i}}$ **(**$\boldsymbol{u}_{\boldsymbol{i}}$**)** |
| --- | --- | --- | --- | --- | --- | --- | --- |
| **False positive (sec)** | 610 | 380 | 386 | 416 | 472 | 366 | 364 |
| **False positive Rate (%)** | 0.1435 | 0.0899 | 0.0908 | 0.0979 | 0.1110 | 0.0861 | 0.0856 |

**Supplementary table 1.** The FP of proposed features in the initial seizure ($\vec{e_{i}}$) versus the whole seizure ($\vec{e_{w}}$) segments.
The table summarizes the rates of FP from non-seizure data set for 5-days (425,100 sec). Non-seizure data include inter-ictal spikes, electrical and motion artifacts as represented in supplementary figure 1. The results shows that the proposed features had minimal rates of FP, which was comparable in the initial seizure versus the whole seizure segments.
