# Supplementary material for: Early Seizure Detection by Applying Frequency-Based Algorithm Derived from the Principal Component Analysis
Source: Front Neuroinform. 2017 Aug 17;11:52. doi: 10.3389/fninf.2017.00052 (PMC5562675; doi:10.3389/fninf.2017.00052)
Supplement: Supplementary file 2 [file Image1.PDF]

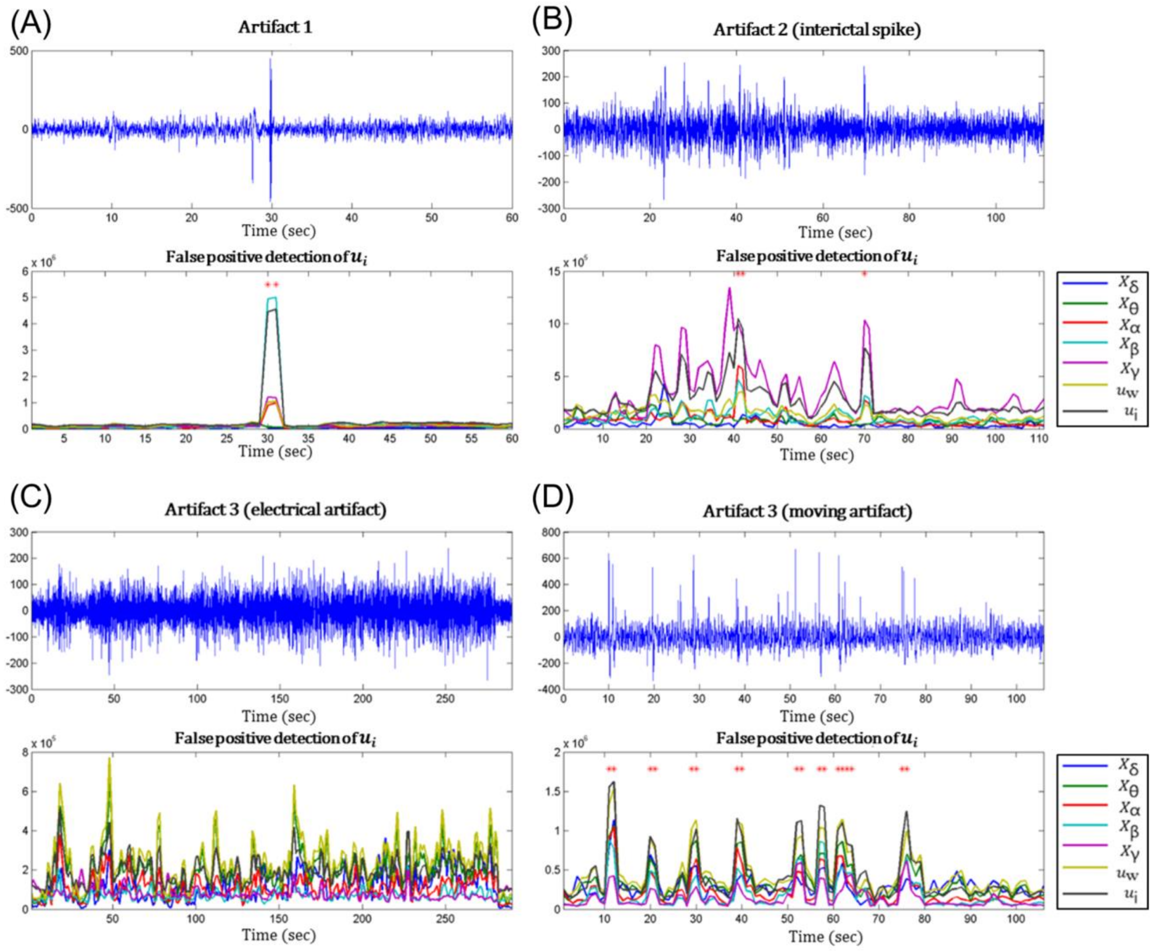

Supplementary figure 1. Examples of false positives from additional non-seizure data set including interictal spikes (A, B), electrical artifacts (C), and moving artifacts (D).
The y-axis represents the feature vector normalized against threshold, therefore, the value larger than 1 represents false positive. The sky-blue, orange, yellow, purple, green, bold blue, and bold red lines denote the signal energy of the delta (δ), theta (θ), alpha (α), beta (β), gamma (γ), the PCA based feature from the initial segment of seizure segment (u_i), and the other PCA based feature from the whole seizure (u_w), respectively. The red dots in the plot show the result of the detection as seizure. In other words, in the non-seizure segment, the red dot means the false positive.
