# Supplementary material for: Early Seizure Detection by Applying Frequency-Based Algorithm Derived from the Principal Component Analysis
Source: Front Neuroinform. 2017 Aug 17;11:52. doi: 10.3389/fninf.2017.00052 (PMC5562675; doi:10.3389/fninf.2017.00052)
Supplement: Supplementary file 3 [file Image2.PDF]

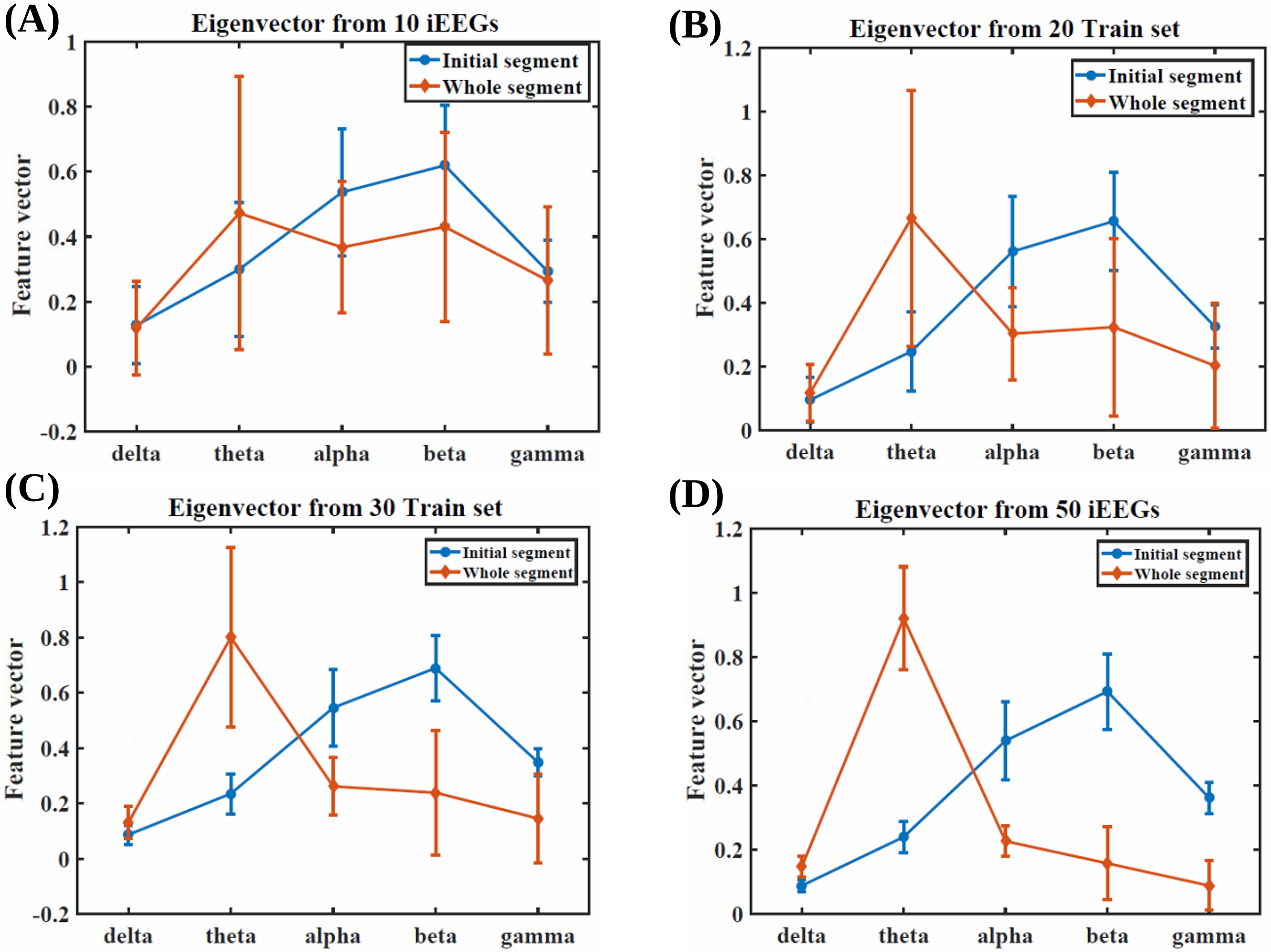

Supplementary Figure 2. Eigenvectors derived from the initial seizure vs the whole seizure segments according to the size of training dataset with 10 (A), 20 (B), 30 (C) and 50 (D) ictal EEG segments. The average of the PCA-based eigenvectors shows similar characteristics regardless of the sample size; however the standard deviation increases as the sample size decreases. The overall trend of eigenvectors from the initial seizure segments shows similar findings across different numbers of the training dataset - the highest weight is on the beta band followed by the alpha band and the lowest weight is on the delta band. On the other hand, those from the whole seizure segments elucidate that the larger numbers of the training dataset, the more evident characteristics of the weight on the five subbands are, especially in the theta and beta bands.
